# A qualitative exploration of triggers for alcohol use and access to support during the COVID‐19 pandemic among people identifying as problem drinkers in the United Kingdom

**DOI:** 10.1111/dar.14013

**Published:** 2025-02-11

**Authors:** Hadia Yaqubi, Tom May, Alexandra Burton

**Affiliations:** ^1^ University College London Medical School London UK; ^2^ NIHR Health Protection Research Unit in Behavioural Science and Evaluation University of Bristol Bristol UK; ^3^ Institute of Epidemiology and Healthcare, Department of Behavioural Science and Health University College London London UK

**Keywords:** alcohol drinking, COVID‐19, qualitative research

## Abstract

**Introduction:**

A polarisation of drinking behaviour was observed during the coronavirus disease (COVID‐19) pandemic, with some people reported to be drinking more alcohol and others less. We aimed to understand how and why the COVID‐19 pandemic and associated restrictions impacted alcohol use and access to support and services during this time.

**Methods:**

We conducted semi‐structured qualitative interviews with 27 participants, including 20 people identifying as problem drinkers and seven alcohol service providers. Data were analysed using thematic analysis.

**Results:**

We identified two main triggers for alcohol use during the pandemic: (i) loss of daily routine and activity resulted in drinking to cope with social isolation and boredom; and (ii) drinking alleviated feelings of fear, anxiety and anger over the imposition of pandemic restrictions. Regarding access to services, two main themes were generated: (i) remote service provision was perceived as inferior to in‐person services; and (ii) the need to offer choice and flexibility in how services were provided, with service providers reporting more positive experiences of online and telephone service delivery than service users.

**Discussion and Conclusions:**

This study provides new insights into potential triggers for alcohol use among people identifying as problem drinkers during the COVID‐19 pandemic. The acceptability of remote forms of service provision were dependent on service user access to, and comfort with using technology. Hybrid delivery models may therefore be suitable in some but not all circumstances, and efforts should be made to promote equitable access to services.

## 1 INTRODUCTION

In March 2020, a national United Kingdom (UK) lockdown order in response to the coronavirus disease (COVID‐19) pandemic led to growing concerns about how the general population would cope with social isolation and other COVID‐19 related changes. Lockdown measures extensively affected the daily lives of individuals through restrictions on leisure activities and work environments and a reduction of in‐person social interaction [1]. There is now a broad consensus of a link between stringent lockdown measures and poorer mental health [2]. One UK‐based longitudinal study found that depression and anxiety levels were highest during the early stages of lockdown, but declined rapidly as restrictions were eased [3].

During the initial stages of the pandemic, a decline in participation in healthy lifestyle behaviours was also observed, including unhealthy eating and reduced physical activity [4]. The UK Health Security Agency, however, reported a polarisation in drinking habits, with increased alcohol use among some participants and less consumption than before the pandemic among others [5]. Comparable patterns have been observed in Canada [6], the USA [7, 8], Poland [9] and Australia [10]. Systematic reviews conducted across 58 countries revealed considerable heterogeneity in overall alcohol consumption, both within and across countries, relative to pre‐COVID levels [11, 12]. Another meta‐analysis examining alcohol use during the COVID‐19 pandemic in Europe revealed that more individuals reduced their alcohol consumption than increased it, with a decline in drinking frequency, quantity and heavy episodic drinking; however, individuals with previously high alcohol use tended to increase their consumption [13]. Cultural and contextual differences may affect alcohol use patterns, and findings appear to differ across settings.

People who reported drinking more during the pandemic were therefore more likely to already be heavier drinkers and were from the most socio‐economically disadvantaged households [14]. In 2020, increases in alcohol consumption were related to higher mortality rates and alcohol‐associated liver disease [15], with the highest annual number of alcohol‐specific deaths reported in the UK since 2001 [16]. A decline in alcohol‐specific UK hospital admissions was also observed at the start of the pandemic, which may have been due to people avoiding hospitals because of fear of COVID‐19 infection or to ease perceived pressure on the National Health Service [16, 17, 18].

Alcohol use has long been identified as an ‘avoidant’ coping strategy [19] used in times of psychological distress, loneliness and adverse life experiences, including trauma and financial adversity. This behaviour is particularly prevalent among those experiencing economic hardship, anxiety and low self‐esteem [20]. Many of these factors were amplified by pandemic conditions alongside new pandemic‐related stressors, such as anxiety around catching COVID‐19 and the perceived health consequences of infection [21]. Factors such as having children at home who should have otherwise been at school or nursery and reduced contact with family and friends, were identified as predictors of coping‐motivated drinking, which in turn was associated with increased alcohol use and problem drinking [22]. Negative coping strategies associated with alcohol consumption may also exacerbate post‐traumatic stress over time [23].

A qualitative study conducted in Australia revealed that females rationalised increases in alcohol consumption in response to dealing with the uncertainty of the pandemic, to cope with sudden changes in work, study or social routines and that they had lower perceived risks of alcohol harm during this time [24]. Similarly in the UK, young women described changes to their work, social lives and living situations as influential for alcohol use [25]. Other qualitative studies conducted in the UK during the first 6 months of the pandemic identified that older adults used alcohol to cope with lockdown, to overcome boredom and as a replacement for social activities or support that was no longer available to them [26, 27]. The ease of purchasing alcohol through online shopping and the desire to recreate social spaces, such as nightclubs or bars, within the home also led to increased consumption among UK adults who self‐identified as regular drinkers [28].

Although qualitative evidence suggests that alcohol consumption may have changed in response to pandemic‐related factors among specific groups of people (e.g., older adults and women), there is limited qualitative exploration as to why alcohol use may have been impacted during this time among a broader demographic, particularly in the UK as the pandemic restrictions continued. There is also limited evidence exploring how support and treatment services were impacted and how changes to service provision and support influenced alcohol use. Qualitative approaches can provide detailed insights into the motivations and triggers of people's drinking behaviour as well as provide an understanding of the types of support made available to them during an unprecedented global event. This study therefore aimed to explore how and why the pandemic and associated restrictions impacted: (i) alcohol use; and (ii) access to support and services for people who identified as problem drinkers.

## 2 METHODS

Semi‐structured individual interviews were conducted as part of the University College London (UCL) COVID‐19 Social Study. The COVID‐19 Social Study was a large mixed‐methods study that aimed to explore the social, psychological and behavioural impacts of the COVID‐19 pandemic in the UK through a longitudinal survey and qualitative interviews with sub‐groups of people whose health and well‐being may have been impacted more adversely by the pandemic (e.g., due to pre‐existing health conditions, key worker status, socioeconomic factors and/or substance misuse problems) [29]. The UCL Research Ethics Committee approved the current study (references: 14895/005, 6357/002).

### 2.1 Sample and recruitment

Participants were eligible for the study if they were living in the UK and aged 18 or over. For the alcohol user group, participants self‐identified as having current or recent experience of problem alcohol use; either reporting pre‐existing problematic drinking behaviours before the pandemic, or, drinking behaviours that had become problematic during the pandemic. Service providers were eligible for the study if they were employed by, or volunteering with a third sector support service for people experiencing alcohol problems.

Participants were recruited via a study poster circulated via social media and shared by community organisations and third‐sector alcohol service providers with their staff and service users. Participants were directed to contact a member of the research team if they were interested in the study and if they perceived themselves as eligible to take part. They were then emailed or given the information sheet and consent form and encouraged to contact the researcher with any questions before signing the consent form. Once the consent form had been received, participants then completed a demographics questionnaire, including their age, gender, ethnicity, living arrangements, relationship and employment status. The researcher then arranged a time to conduct the interview.

### 2.2 Interview and transcription procedures

Interviews were conducted between June 2021 and February 2022. TM or AB conducted interviews online via Microsoft Teams; however, three interviews were conducted in‐person within third sector alcohol services at the request of the participants. Social distancing rules at the time of the in‐person interviews were followed, such as the interviewer wearing a mask and conducting the interview in a well‐ventilated room. The interviews followed a semi‐structured topic guide designed to illicit responses on the impact of the pandemic on alcohol use, mental health and well‐being and the provision of support and services (see Supporting Information S1 and S2). Participants were offered a £10 voucher as compensation for their time. Interviews lasted between 23 and 79 minutes (average 42 minutes) and were audio recorded and transcribed by a UCL‐approved external transcription company. All identifiable information, including names of people, services and locations were redacted from the transcripts before analysis.

### 2.3 Data analysis

Data were analysed using thematic analysis [30] guided by the six‐step process proposed by Braun and Clarke [31]. Our research methodology was informed by critical realist ontology [32], whereby we sought to identify key triggers for alcohol use and factors that impacted access to services through an in‐depth exploration of participant accounts and experiences. For participants, the analysis focused on their own experiences of: (i) how the pandemic impacted their alcohol use; and (ii) their ability to access services. For service providers, the analysis focused on: (i) how they perceived the pandemic had impacted the alcohol use of existing or new clients; and (ii) their client's ability to access services. Transcripts were imported into NVivo12 software for analysis. An inductive analysis approach was taken with TM and HY reading each transcript to familiarise themselves with the content and then conducting line‐by‐line coding of the transcripts with particular attention to passages of text that were relevant to the research questions. Code names were developed so that they closely aligned with participant's own experiences and words. Codes were then grouped into similar topics, and patterns across participant accounts were identified. The research team met fortnightly to discuss emerging analyses and codes and to agree on the final themes and subthemes.

## 3 RESULTS

### 3.1 Participants characteristics

We interviewed seven alcohol service providers and 20 people who self‐identified as either: (i) long‐term dependent drinkers; and (ii) those with no previous history of problem alcohol use who considered themselves as social drinkers before the pandemic and reported increased harmful or problematic alcohol use during the pandemic. Four of the 20 participants were recruited via third sector community services providing support to people experiencing problems with alcohol and four participants were recruited via third sector drug and alcohol services. The remaining 12 participants were recruited through social media. Participants were aged between 40 and 66 years old (average age 52) and predominantly female, White British, single, divorced or separated. Service providers were predominantly female with over 10 years' experience working in alcohol support services (Tables 1 and 2).

**TABLE 1:** Characteristics of participants with drinking experience.

Demographics	Range (mean)/*N*
Age, years	40–66 (52.5)
Gender	
Female	12
Male	8
Ethnicity	
White British	13
White other	3
White Irish	2
Black or Black British Caribbean	1
White and Asian	1
Employment status	
Unable to work due to disability/illness	7
Full time employment	5
Retired	3
Unemployed and seeking work	2
Part time employment	1
At university	1
Self‐employed	1
Living status	
Live alone	11
Live with others	9
Relationship status	
Single	9
Divorced or separated	7
Married or co‐habiting	4
Drinking status	
Long‐term dependent drinkers	15
Social drinkers	5
Service user before the pandemic	
Yes	10
No	10
Service user during the pandemic	
Yes	9
No	11^a^

^a^ One participant attempted to access services during the pandemic, but was refused access.

**TABLE 2:** Characteristics of service providers.

Demographics	Range (mean)/*N*
Age, years	31–51 (40.7)
Gender	
Female	6
Male	1
Occupation	
Recovery coordinator	3
Team leader	2
Engagement worker	1
Lived experience volunteer	1
Years of experience working in alcohol services	
0–1 years	1
2–3 years	1
4–5 years	1
10 years +	4
Location	
West Midlands	3
Yorkshire and Humber	3
London and Southeast	1

### 3.2 Themes

Two main triggers for increased use of alcohol during the pandemic were identified: (i) loss of daily routine and in‐person activity; and (ii) fear and anxiety about following pandemic rules. Two main themes were also generated around access to services: (iii) remote service provision experienced as inferior to in‐person services; and (iv) the need to offer choice and flexibility in how services were provided. Themes and subthemes are summarised in Table 3 and described in detail with accompanying quotations from participants below. All participant and service provider names have been replaced with an anonymised identification number after each quote.

**TABLE 3:** Summary of themes and subthemes.

(1) Loss of daily routine and in‐person activity
Changes to working lives and impact of homeworking
Social isolation following the loss of in‐person socialising and activities
Escapism from boredom
(2) Fear and anxiety about following pandemic rules
(3) Remote service provision experienced as inferior to in‐person services
Breakdown in communication and monitoring between service users and services
Therapeutic encounters conducted remotely at home
(4) The need to offer choice and flexibility in how services were provided

#### Loss of daily routine and in‐person activity

Participants described how social restrictions created by the pandemic altered their pre‐existing ‘problematic’ drinking habits or initiated new patterns of increased drinking due to a change in routine and a reduction in in‐person activities. Self‐reported triggers of problematic alcohol use were primarily linked to a change in working lives and a decrease in accountability that accompanied homeworking, as well as feelings of social isolation and loneliness due to the breakdown of in‐person support systems and activities.

##### Changes to working lives and impact of homeworking

Participants found that the removal of divisions in the day due to working from home (e.g., using the commute to and from work to decompress) made it difficult to establish a daily rhythm. Subsequently, many participants resorted to using alcohol as a coping strategy to de‐stress and differentiate home life and work and to help them relax and unwind:

‘It was a reward since you're at home all day working, it was a natural break point associated with that finishing work is the “quote, end quote,” reward of being able to unwind with alcohol.’ (P10_male_aged_40–44_social_drinker)

Service providers also reported a marked increase in referrals from people who had experienced a change in employment status, either through furlough or job loss:

‘Lots of people who have been furloughed or lost their job during Covid are drinking, or we've had lots of people who have said, “Yes, during lockdown my alcohol levels have gone up” so alcohol referrals have just gone through the roof.’ (Service_provider_7_aged_30–34)

In contrast, one participant experienced being unable to work during lockdown as a ‘*wonderful chance for my recovery*’ as his working environment was the main trigger for alcohol use:

‘I feel really comfortable with lockdown because I have not got to work, so that can challenge me with alcohol and with the relationship with the people and the relationship with the stress of the job, it's a high pace of work and stress.’ (P4_male_aged_55‐59_long_term_drinker)

##### Social isolation following the loss of in‐person socialising and activities

Participants reported how the psychological impact of social isolation was a trigger for alcohol use and solitary drinking:

‘The busier you are, the happier you are in yourself and content with yourself, the more likely I am not left alone with these dark thoughts and negativity, I like to be kept busy and with the lockdown I haven't been. I spoke with my doctor to get counselling because there's things inside of me that I need to get out, how isolated I felt, I want a drink.’ (P1_female_aged_55‐59_long_term_drinker)

Moreover, the limited opportunities to socialise and meet new people in person, particularly for people living alone, exacerbated feelings of loneliness, which led to increased drinking:

‘I am single, and … that's not been really possible to meet people, so I suppose that has contributed. You start to think, well, is this ever going to go away? I mean if you start to think about all that, it's really depressing. I suppose that part of me, when I start to think like that, I think, well, what's the point? I might as well have a drink.’ (P12_female_aged_40–44_long_term_drinker)

Social distancing and lockdown measures also drastically reduced opportunities for participants' to be held accountable and monitored by their friends and family, which facilitated increased consumption:

‘I thought, do you know what? If I had a drink during lockdown—this was the beginning of it—I can have a few drinks. No one will know because no one will come round, and no one will see me. And that will be all right.’ (P11_female_aged_40–44_long_term_drinker)

The positive impacts gained from attending in‐person community activities were a core motivation to actively engage with those activities. The subsequent transition to online interactions dampened participant motivation to interact with others. Some participants reported self‐medicating with alcohol following their inability to take part in protective in‐person activities:

‘I just think it had all got on top of me really. Stress. Not being able to see anybody. Not being able to go places. Not do swimming. Not do gardening. And I just thought, I'd had enough. And I went out and I had two bottles of wine within about 20 minutes.’ (P6_female_aged_60–64_long_term_drinker)

Alcoholics Anonymous (AA) meetings were an important source of in‐person support before the pandemic for many participants. Consequently, many participants had difficulty adjusting to the reduced accessibility to this service:

‘I mean [AA] was also like a big thing in the early part of lockdown just because it's always in my mind like, oh if things are getting really bad, I can just go to a meeting and then that will help, and then that was taken away.’ (P3_female_aged_45–49_long_term_drinker)

##### Escapism from boredom

Boredom resulting from the closure of in‐person community support systems, a lack of routine and being stuck at home led to an increase in alcohol use with many participants stating they used alcohol as escapism from ‘*being a bit stuck*’ and experiencing ‘*the same thing every day*’:

‘Theres no services open, there's no classes open. I used to do an art class, that's all closed. It's total boredom, you're sitting at home and you're so bored. And so, I would say that's why you find something to do, just wanting the time to tick away and drinking and/or using helps to pass the time.’ (P15_female_aged_55–59_long_term_drinker)

‘The boredom ties back to the drinking thing, especially during the summer. I was bored, it was hot, it was bright, I couldn't sleep so that is when I would drink.’ (P5_female_aged_50–54_long_term_drinker)

#### Fear and anxiety about following pandemic rules

Participants described using alcohol to control their anxiety around inadvertently breaking social distancing rules. This often occurred when they needed to provide care and support to unwell relatives, or gain support from friends or family to protect their own mental health:

‘When it was lockdown, I still went round [name of son's] two, three times a week for both our mental health. He had Covid at the beginning, he was really ill … He almost went because he struggled to breathe, and I had to have my grandchild for two weeks and every time I went anywhere, I was looking over my shoulder thinking I was going to get arrested for breaking Covid [rules]. It was horrible …’ (P8_female_aged_60‐64_long_term_drinker)

Some participants also described drinking or craving alcohol in response to fear and anxiety about feeling trapped and controlled by government rules and experiencing a lack of agency because the restrictions ‘*were imposed on us*':

‘It's the social, in terms of the curtailment of freedom I'm most concerned about. I'm not vaccinated and probably will not get vaccinated and obviously then that maybe mandates, which will prevent me from doing certain things and that's always a concern for me.’ (P14_male_aged_50‐54_social_drinker)

Observing members of the public breaking the rules also triggered alcohol use for some participants to control their anger or frustration:

‘[My neighbour] had people in and out during the first lockdown when I think everybody was … sticking to the rules and I just lost my top one day. I thought it was completely irresponsible. I had a lady that I'd met … she was an intensive care nurse and I'd have her on the phone crying, and it'd just be like what are these people upstairs doing? They are having all these people in … I was so angry about it all … I would get annoyed and then I'd drink on it and then I'd get more annoyed.’ (P5_female_aged_50‐54_long_term_drinker)

#### Remote service provision experienced as inferior to in‐person services

For participants who received support from formal services before the pandemic, the rapid shift from in‐person to remote contact appeared to jeopardise engagement with treatment. Participant narratives indicated a greater potential for miscommunication between service providers and service users during remote, compared to in‐person appointments and a preference for face‐to‐face interactions. The removal of in‐person monitoring also strained relationships between service users and keyworkers and increased the potential for alcohol misuse. Barriers to connectivity (e.g., digital exclusion of vulnerable groups lacking technology skills or resources) influenced the extent to which participants felt able to seek support. A further barrier to engagement was the lack of private physical space for participants to occupy during therapeutic encounters; however, a small number of participants and most service providers welcomed the flexibility of being able to access online support.

##### Breakdown in communication and monitoring between service users and services

Many service providers could only offer telephone appointments during the initial stages of the pandemic, which impacted their ability to interact and engage effectively with clients:

‘We had to think differently because (you) don't have things like your communication skills, you don't have your body, it's just gone, it's all on the telephone, so you've really got to have amazing listening skills to pick up things.’ (Service_provider_4_aged_40–44)

The lack of in‐person appointments enabled some participants to conceal the extent of their alcohol use, citing a lack of accountability to services for their increased alcohol consumption which negatively impacted their overall health:

‘There wasn't anybody there … because there wasn't any telephone calls and I didn't have to report in, I wasn't uploading any of my blood sugar levels so in my mind … it's almost like if you can't see me then I'm safe, so I carried on drinking, knowing what it was doing to me but in another way thinking, Well hey, nobody is saying anything so I'm just going to carry on.’ (P18_male_aged_55‐59_social_drinker)

Service providers also generally stated a preference for face‐to‐face meetings to be able to effectively monitor people's alcohol use and felt less confident in relying on verbal accounts:

‘They could be telling us that they've been sober for X amount of days or weeks but really we don't know. Because even though they may come across as if they've been drinking, we can't accuse them of drinking because we don't know that for sure, it's the uncertainty really. Whereas with the face‐to‐face group, you can see for yourself what's going on …’ (Service_provider_3_aged_45–49)

The same service provider did, however, suggest that online meetings could be a useful assessment mode for service users who may feel more comfortable expressing their support needs in a more anonymous environment:

‘People are opening up more … when they've got the screen to hide behind … because sometimes to be face‐to‐face with somebody might be a bit more difficult, maybe they don't feel as if they could be as vulnerable.’ (Service_provider_3_aged_45–49)

Some participants felt that their lack of ability to adjust to, and subsequently engage with remote service provision due to heightened levels of anxiety and stress, was misinterpreted as indifference by service providers:

‘I got the impression that people thought well we've offered you this and you're not engaging. But they didn't actually want to know the reasoning behind (it) which (was) I didn't want to do zoom meetings.’ (P5_female_aged_50‐54_long_term_drinker)

##### Therapeutic encounters conducted remotely at home

Some participants expressed dissatisfaction or disengaged with remote appointments provided via videoconferencing platforms because they perceived it as an invasion of their private home life:

‘I feel like I'm being invaded, that my home is my safe place … my daughters are like don't be so ridiculous, you can put a white sheet up over your pictures. And I said no, I don't care, I don't want people in my home.’ (P5_female_aged_50‐54_long_term_drinker)

Whilst reports of intimate partner violence were rare, some instances of emotional abuse were described. Controlling behaviours culminated in drinkers having to conceal their alcohol use from their partners making it difficult for them to adhere to treatment plans:

‘They [service] want us to keep a diary, but I can't do that, because if it is found, the other half will go nuts. I can't be honest with him. So, rather than writing a diary, I just hide my alcohol and put the cider in the fridge. Cider's fine as far as he's concerned.’ (P7_female_aged_50‐54_long_term_drinker)

Contrastingly, a small number of participants valued the convenience of online appointments. This was attributed to reduced costs and time needed for travel to and from in‐person appointments. Additionally, online appointments avoided the need to comply with social distancing restrictions associated with in‐person meetings:

‘Because I am at my home, with my things, with my tea, with my cup … So now, AA meeting – there is a queue and after 15 people you can be refused to attend the meeting. So, you have to go one hour in advance … I prefer to stay at home.’ (P4_male_aged_55‐59_long_term_drinker)

#### The need to offer choice and flexibility in how services were provided

Most of the service providers we interviewed acknowledged difficulties in setting up remote services at the start of the pandemic. However, as time went on and procedures became more established, they observed it working well for some clients. They therefore continued to offer a choice of remote or in‐person appointments as social distancing restrictions eased:

‘It took the pandemic for us to realise if somebody wants a telephone call, that's alright. If that suits them and you can deliver an intervention in that way. I think it's taken this big change for us to realise that yes, there are different ways other than just one‐to‐one, face‐to‐face appointments. It's about what the client wants, isn't it? I think as a team we're keeping that element where the client can choose how they have their appointments.’ (Service_provider_4_aged_40–44)

Many participants, however, experienced challenges in effective communication. These difficulties stemmed from a lack of familiarity with accessing services online, feeling more comfortable engaging in‐person than online, or digital connection issues which posed an additional obstacle to engagement:

‘Quite often the lines were bad because I only use the mobile, we haven't got a house phone, and she was obviously doing it from home, and lives in quite a remote area. Like, it got to, well why bother?’ (P6_female_aged_60‐64_long_term_drinker)

Service providers also acknowledged that for some people, online meetings were not appropriate due to lack of confidence, anxiety or difficulties accessing technology:

‘Lots of people don't have the kind of tech to get onto the meetings, so that put a barrier in the way for people accessing specific parts of treatment that would have been beneficial. One‐to‐one appointments all went to telephone … We've had mixed reviews, some people really had success speaking to people on the phone but we've also lost contact with lots of people that we might have seen on site.’ (Service_provider_7_aged_30–34)

To counteract some of the challenges in accessing remote services, one service provider implemented a space for service users to access computers, which enabled them to attend online support meetings:

‘What I've had to do is over the last six or seven months, the hubs have slowly started to open, limited access of the hubs, and they've created rooms in their offices where people can go and use the computers, get support to use their computers, to access the zoom meetings.’ (Service_provider_6_aged_40–44)

Service providers also identified specific groups of people who they struggled to engage with remotely and for whom online appointments might not be appropriate. Service providers felt that it was vital to provide in‐person services to people experiencing homelessness, people transitioning out of hospital and those with complex mental health needs to ensure they remained engaged:

‘People that could only be supported by being seen face‐to‐face are never going to be able to engage with that, so the homeless, people with such high anxiety that they don't answer the phone, they don't have a phone, they're already socially isolated, those are people that you would struggle to engage so trying to only do that by phone is just going to make it worse.’ (Service_provider_5_aged_30–34)

Shift workers were also identified as a group who were difficult to engage remotely, given their unsociable and irregular working schedules:

‘I think it's fair to say that workers, especially shift workers, if they have been working through the pandemic have been quite hard to get hold of because—I mean they always have been, because if their shifts change and so on, we can't get them on the phone, or maybe it's out of hours that they finish, and we're not working, so you end up chasing them.’ (Service_provider_2_aged_35–39)

## 4 DISCUSSION

We explored how the COVID‐19 pandemic and associated restrictions impacted alcohol use and access to services and support among people identifying as problem drinkers. We identified two main triggers for increased alcohol use, including changes to routines and a reduction in daily activities, which led many participants to drink alcohol to cope with social isolation and boredom. We found similar patterns to those identified by a previous qualitative study with UK older adults [26], such as the importance of face‐to‐face interactions for services users, the challenges of social isolation and loss of routine on alcohol use, and the critical need for accessible mental health support in addressing alcohol misuse. Additionally, we found that alcohol was used to cope with fear and anxiety provoked by the implementation of pandemic restrictions.

The shift to remote alcohol service provision introduced barriers to service engagement, including issues of digital exclusion and a preference or necessity for in‐person interactions. Despite these challenges, service providers were more positive in their experiences of remote service delivery and advocated choice and flexibility of appointment mode even once the pandemic had ended. They did, however, acknowledge that remote services might not be appropriate for those with more complex health or social needs.

### 4.1 Pandemic related triggers for alcohol use

Our findings align with other qualitative studies identifying how a sudden shift in working practices and changes in employment status increased alcohol use [25, 32] and that pandemic‐related job loss and financial insecurity increased domestic alcohol consumption to cope with uncertainty and stress [25, 33]. Our research also highlights, however, that drinking patterns were influenced by increased home working, changes in work–life routines (e.g., temporary furlough) and blurred boundaries between personal life and work. A previous longitudinal survey found that similar changing contexts and situations (e.g., more free time/boredom) facilitated more frequent consumption [34]. It is therefore important that services recognise how changes in consumption and patterns of alcohol use, including increases in frequency and duration of drinking periods, can be instigated by both external stressors as well as changing contexts and environments prompted by isolation periods. While the continuation of flexible working post‐pandemic may improve work–life balance for many, employers need to be mindful that this approach may not be universally beneficial; for some, this could lead to a continuation or worsening of alcohol‐related issues [35]. To support employees navigating these complexities, initiatives that encourage hybrid approaches, face‐to‐face or regular contact between employees and teams and access to mental health support services may enhance overall well‐being and meet diverse workforce needs.

As well as the loss of work, the loss of social connections was found to be a risk factor for increased alcohol consumption during the pandemic. For some, the psychological impact of isolation, including increased feelings of loneliness, acted as a trigger for increased alcohol use. While social isolation and loneliness during the pandemic have been widely cited as a risk factor for increased alcohol consumption [32], we also observed how lockdown measures meant that peers and other social network members were unable to influence and moderate increased patterns of domestic drinking. There is evidence that social connections can play a crucial role in moderating alcohol consumption by buffering poor mental health [36], and research conducted before the pandemic has highlighted the importance of social support for reducing alcohol use [37]. Self‐regulation is also known to play a key role in controlling alcohol use [38]. A previous qualitative study found that Danish and Australian women aged 50–70 often used social connections to set informal limits and self‐regulate their drinking [39]. Our findings suggest that the absence of social cues during lockdown may have led to more excessive drinking, especially for those who rely on their social circles for support and moderation. Hence, the inclusion of peer support measures in future alcohol services and support for those with limited social networks might help limit the negative impacts of social isolation and loneliness more generally on domestic consumption.

Fear and anxiety about following pandemic rules was found to have a detrimental impact on alcohol consumption among our participants. This mirrors findings from our previous work that showed how concerns about understanding and following the rules, as well as the behaviour of others breaking the rules negatively affected mental health and well‐being [40, 41, 42]. For participants in the current study, however, alcohol was actively used as a strategy to cope with daily anxiety and frustrations encountered during this period. This highlights the importance of measures that allow for some flexibility among people identifying as problem drinkers, who may benefit for example, from extended support bubbles. Co‐located services that address both mental health and problematic alcohol consumption have been suggested as effective interventions in similar contexts [43, 44]. Despite the recognised need, integrating mental health and substance use services remains challenging due to institutional separation, differing treatment philosophies and inadequate collaboration, often leaving individuals with complex needs underserved [45].

### 4.2 Implications for the future delivery of alcohol services

Changes in alcohol consumption and service access during the COVID‐19 pandemic remain relevant in a post‐pandemic context. Our findings show that the pandemic intensified triggers such as stress, anxiety, social isolation, and disrupted routines, which influenced drinking behaviours. These triggers affected individuals' alcohol use and service engagement, making it crucial to understand their long‐term impacts beyond the pandemic and how best to support people going forward.

Our work uncovered a discrepancy between service users and providers regarding their preferences for the future provision of services. While providers often highlighted the flexibility and convenience offered by remote consultations, there were concerns about the effectiveness of monitoring service users' alcohol use through such means. Conversely, most service users expressed a preference for in‐person consultations, viewing remote services less favourably. The difference in response is not uncommon: existing research has shown how client contexts, including access to technology, technical issues (e.g., broadband quality) and privacy concerns function as a barrier to remote service provision [46]. Although the provision of accessible private rooms located in services and the distribution of mobile phones have proved successful in improving uptake of telehealth substance use services [47, 48], there remains a need to ensure that access to remote services becomes more equitable, especially as their use expands post‐pandemic [46]. These findings have implications for the design and delivery of alcohol support services in a post‐pandemic world. The challenges associated with remote service provision highlight the need for flexible, hybrid models that accommodate diverse user needs and circumstances.

Therapeutic alliance is crucial for successful addiction treatment outcomes [49]. Previously established relationships between service users and service providers enabled some service users to continue to receive support remotely. This aligns with previous literature highlighting the importance of continuity of care in establishing trust in addiction treatment [50]. However, there was evidence that some service users—particularly those who had not accessed services before the pandemic—encountered difficulties in establishing and maintaining relationships with service providers remotely. These difficulties were attributed to the potential for miscommunication and the increased ability to conceal alcohol use from services that jeopardised positive outcomes. These findings also highlight the importance of providing choice of treatment modality, including hybrid approaches in the future delivery of services. Participant preference, as well as the context and purpose of engagement, all need to be considered. Remote services might prove beneficial for routine appointments involving individuals familiar with services, while in‐person appointments may be more suitable for first‐time attendees, or those at a high risk of disengagement or alcohol‐related harm, such as withdrawal or relapse. However, while service user choice in modality can promote autonomy and subsequent engagement [46], our findings also indicate that service providers still expressed a preference for face‐to‐face over online interactions to be able to effectively monitor people's alcohol use. Hence, there may still be a need to restrict the mode of treatment based on the specific context and circumstances of the service user.

### 4.3 Strengths and limitations

This UK qualitative interview study includes both service provider and problem drinker experiences, enabling a more nuanced understanding of the social and psychological effects of the COVID‐19 pandemic on individuals identifying as problem drinkers. The study was conducted when COVID‐19 social distancing measures were relaxed; therefore, participants were able to participate in‐person if they preferred, overcoming some of the digital barriers to engagement identified in our research. The study does, however, have limitations; first, we did not conduct alcohol screening for inclusion in the study and relied on people self‐identifying as problem drinkers. This may have excluded those who were unaware or who did not want to acknowledge problematic alcohol use, and their experiences may have been different from those represented here. Second, only a third of our problem drinker sample were in employment, with most identifying as office workers who transitioned to working from home. While our sample therefore reflects previous quantitative findings that people who reported drinking more during the pandemic were more likely to be economically inactive [6], we did not obtain the views of other economically active, but potentially disadvantaged groups, such as shift or key workers. These groups may have experienced different triggers for alcohol use and additional challenges in accessing support during this time. Third, a higher number of female alcohol users took part in this study than males, which typically does not represent the gender profile of alcohol users in the UK. Participants were also middle aged; younger or older people may have had different experiences of alcohol use and access to support during the pandemic. Lastly, only 9 of the 20 participants engaged with alcohol support services during the pandemic, and one participant tried to engage, but was advised not to by services. This limits the applicability of these specific findings to a smaller subset of our sample; however, we also aimed to understand access to more informal types of support and how this impacted drinking behaviours across the spectrum of long‐term dependent and social drinkers.

## 5 CONCLUSIONS

This study provides insights into potential triggers for alcohol use during the COVID‐19 pandemic, including common stressors, such as job loss and financial insecurity, as well as changing contexts and routines. These insights are relevant for both individuals who self‐identify as long‐term dependent drinkers and those whose drinking was impacted by pandemic‐related restrictions. If services are to continue with remote provision post‐pandemic, such measures should consider providing additional forms of support that attend to context‐specific features that may trigger changes in drinking patterns. Given that the acceptability of remote provision was dependent on service user access to and comfort with technology, hybrid models may be suitable in some, but not all, circumstances, and efforts should be made to promote access and ensure equity among service users.

## AUTHOR CONTRIBUTIONS

AB and TM conceived and designed the study. TM and AB were responsible for data collection. HY, TM and AB performed the data analysis and HY wrote the final manuscript. AB and TM assisted with reviewing and editing. All authors contributed to manuscript revision, read, and approved the submitted version. Each author certifies that their contribution to this work meets the standards of the International Committee of Medical Journal Editors.

## FUNDING INFORMATION

The COVID‐19 Social Study was funded by the Nuffield Foundation (WEL/FR‐000022583), but the views expressed here are those of the authors. The study was also supported by the MARCH Mental Health Network funded by the Cross‐Disciplinary Mental Health Network Plus initiative supported by UK Research and Innovation (ES/S002588/1), and by the Wellcome Trust (221400/Z/20/Z). The funders had no role in study design, collection, analysis and interpretation of data or in writing the manuscript.

## CONFLICT OF INTEREST STATEMENT

The authors declare no conflict of interest.

## Supporting information


**Data S1.** Interview topic guide: alcohol users.

**Data S2.** Interview topic guide: third sector alcohol service providers.

## Data Availability

The data presented in this article are not readily available because they contain information that could compromise the privacy of research participants. Requests to access the data should be directed to a.burton@ucl.ac.uk.
