# Supplementary material for: A qualitative exploration of triggers for alcohol use and access to support during the COVID‐19 pandemic among people identifying as problem drinkers in the United Kingdom
Source: Drug Alcohol Rev. 2025 Feb 11;44(3):858–70. doi: 10.1111/dar.14013 (PMC11886483; doi:10.1111/dar.14013)
Supplement: Supplementary file 1 — Data S1. Interview topic guide: alcohol users. [file DAR-44-858-s002.docx]

**Interview topic guide: Alcohol Users**

1. **INTRODUCTION**

- **Ask to describe ‘normal life’** (PROMPTS: employment, living situation, education, full time parent/carer, use of community services, pre-existing health conditions)

1. **SUBSTANCE USE**

- **Please describe your current substance use** (PROMPTS: preferred drink, frequency, quantity, period of time of problem use)
- **Was there any impact of the pandemic on your substance use?**
- **Are you receiving any treatment for your substance use?**
- **Was there any impact of the pandemic on your ability to receive/access treatment/support?**

1. **SOCIAL LIFE**

- **How would you describe your social life before the Covid-19 pandemic?** (PROMPTS: social network, social activities, community services/participation, social support)
- **How would you describe your social life now that social distancing measures have been brought in because of Covid-19? Please tell us about this** (PROMPTS: social network, social activities, community services/participation, social support, impact of alcohol use on social life/networks)

1. **MENTAL HEALTH**

- **How do you feel about the changes that have been brought about by Covid-19?**
- **Have they had any impact on your mental health or wellbeing? Please tell us about these** (PROMPTS: what are the things bothering you most, any impacts on positive emotions, impact on sense of identity, negative psychological feelings, physical symptoms due to stress or anxiety, impact of alcohol use on mental health or wellbeing)
- **Have you been doing/ planning anything to help with this?** (PROMPTS: connecting with others, online groups, hobbies, exercise, community engagement/volunteering, anything else)
- **Why are you doing/ not doing these things?**

1. **PROSPECTION**

- **Has the pandemic meant that you have any worries for the future?**
- **How are these different from the worries you had before?**
- **Will this change the way you live your life in future?**
- **Has this changed any of your priorities for the future?**
