# Supplementary material for: A qualitative exploration of triggers for alcohol use and access to support during the COVID‐19 pandemic among people identifying as problem drinkers in the United Kingdom
Source: Drug Alcohol Rev. 2025 Feb 11;44(3):858–70. doi: 10.1111/dar.14013 (PMC11886483; doi:10.1111/dar.14013)
Supplement: Supplementary file 2 — Data S2. Interview topic guide: third sector alcohol service providers. [file DAR-44-858-s001.docx]

**Interview topic guide: Third Sector Alcohol Service Providers**

1. **INTRODUCTION**

- **Ask to describe work/type of service** (PROMPTS: Type of service, clients you work with, what is your role, what services/practices/initiatives you offer)

1. **IMPACT ON CLIENT GROUP**

- **What are the key issues facing your client group during the pandemic?**
- **What has been the impact of the pandemic on your clients being able to access services?** (PROMPTS: service appointments, access to support staff, treatment plans)
- **Have you noticed any impact on their physical/mental health? Could you describe some examples?** (PROMPTS symptoms/side effects, presentation of condition, ability to manage physical/mental health)
- **Have your clients had any difficulties accessing daily provisions** (PROMPTS: sanitation, hygiene products, food) Could you describe some examples?
- **Has anything helped them to access these provisions?**
- **Have you noticed any changes in patterns of substance use? (**PROMPTS: switch to alternative substances/beverages, If so, why? frequency/quantity/ cost? Methods of purchasing/obtaining?

1. **CHALLENGES TO OPERATIONAL PRACTICES/SERVICE DELIVERY**

- **What are the key challenges in delivering your service during the pandemic?** (PROMPTS: Ability to follow social distancing guidelines/maintain delivery of service, changes due to guidelines, safety of staff/service users, maintaining contact/relationships with service users)
- **Has your service made any adaptations in response to the challenges encountered during the pandemic?** (PROMPTS: changes to current practice, new initiatives, methods of communication, location of appointment (e.g. online/telephone), any benefits from the changes

1. **PROSEPECTION**

- **Has the pandemic meant that you have any worries for the future for your client group?**
- **How are these different from the worries you had before the pandemic?**
- **Will this change the way you deliver your service in the future?**
- **Has this changed your priorities as a service for the future?**
